# Using PyMOL to Understand Why COVID-19 Vaccines Save Lives

**DOI:** 10.1021/acs.jchemed.2c00779

**Published:** 2023-02-28

**Authors:** Celia Maya

**Affiliations:** Instituto de Investigaciones Químicas (IIQ), Departamento de Química Inorgánica and Centro de Innovación en Química Avanzada (ORFEO−CINQA), Consejo Superior de Investigaciones Científicas (CSIC), Avenida Américo Vespucio 49, 41092 Sevilla, Spain; Facultad de Química, Universidad de Sevilla, Aptdo 1203, 41071 Sevilla, Spain

**Keywords:** COVID-19 Infection Process, COVID-19 Vaccines, Protein Structure, PyMol Workshops, Function−Structure Relationship

## Abstract

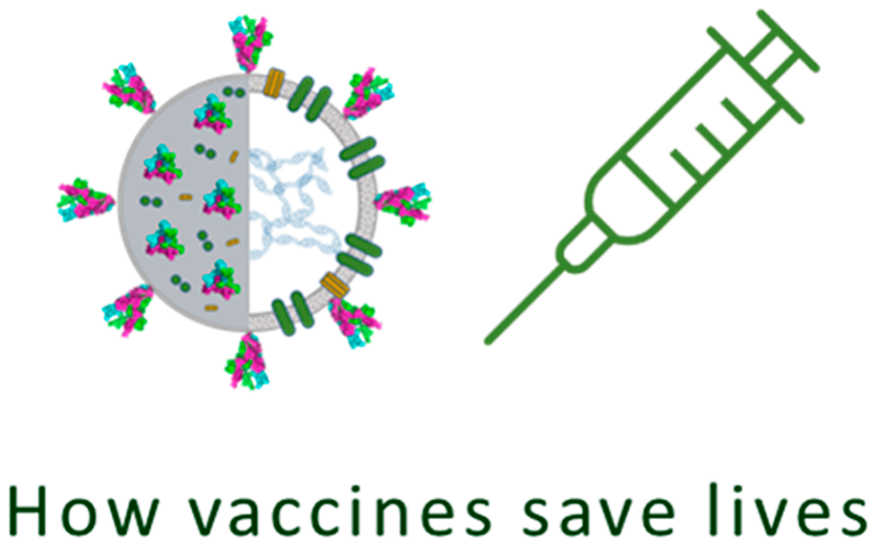

Chemistry and biochemistry instructors must help students to develop the ability to visualize and manipulate 3D biomolecular structures and critically analyze them and their relationship to their functions. To do this, representative systems must be strategically selected to stimulate students’ motivation. Since the World Health Organization declared a global pandemic caused by a new beta-coronavirus, called SARS-CoV-2 in early 2020, huge efforts are being taken by researchers to learn in depth how this virus works and a lot of scientific results are continuously reported. Many of them focus on the structural features of the viral spike glycoprotein and their relation with the vaccine development. This paper presents a series of workouts that deep into the structural characteristics of the spike protein S SARS-CoV-2 virus and the structural features involved in its infection process, using free online resources such as the PDB and the computer program PyMOL. This type of activity is intended to engage structural biology students in examining these macromolecules and others to help establish procedures for controlling COVID-19 and other future infectious diseases. PyMOL session files and student activities are provided.

## 1 Introduction

Proteins and protein complexes are molecular machines that carry out a large number of essential functions in the cells.^1^ Protein functions are directly related to the structures of these proteins. Understanding how these molecules fold, how they assemble into complexes, and how they function could give us clues to answer questions such as why we have cancer, why we get sick, why we grow old, or how we can find cures for many diseases. Fundamental principles of protein folding and assembly, therefore, are an important part of most introductory-level biology and biochemistry courses. Many instructors use molecular visualization tools that allow their students to manipulate protein 3D structures and achieve a better understanding of the structure–function relationship.

Advances in techniques for structure determination of biomolecules such as X-ray crystallography and nuclear magnetic resonance, and, in recent times, cryogenic electron microscopy, have allowed the resolution of more than 190,000 three-dimensional protein structures. All of them are freely available to be examined in the Protein Data Bank.^2^

On the other hand, numerous studies have reported that emotions play a crucial role in the human cognitive processes,^3^ including attention,^4^ learning and memory,^5^ reasoning, and problem-solving. That is why it is very important to select examples that students perceive as interesting and worth knowing, which occurs when learning is connected to students’ interests, aspirations, and life experiences. Nowadays, learning facts about SARS-CoV-2, the coronavirus that causes COVID-19, is a very interesting topic for students. They look forward to understanding how this virus infects cells, how vaccines and antibodies work, or how the efforts of our research scientists can help end the current global health crisis.

In the last two years, hundreds of structures of the SARS-CoV-2 spike protein have been reported. Their analysis has revealed aspects of its structural flexibility and how this protein interacts with the cellular receptor ACE2, revealing the way the immune system prevents its action by blocking it with neutralizing antibodies.

Herein, a COVID-based learning activity is provided to train students in visualization and critical analysis of protein structures using PyMOL software.^6^ An example based on the CoV-2 spike glycoprotein and its interaction with ACE2 and different antibodies is provided. Simultaneously, they are educated in how scientific knowledge is achieved and how it helps to satisfy many basic human needs and improve living standards.

Although targeted at college chemistry, biochemistry, and biophysics students, these activities may be appropriate at the high school level as well, particularly in biology or chemistry courses.

## 2 Methodology (Classroom Activities)

The participants in these activities were 60 fourth-year university-level students from Chemistry and Chemistry & Material Sciences areas. They were separated into two groups that followed the same activities. During the sessions, the 30 subjects shared the same classroom and were instructed by the same teacher. A survey conducted at the beginning of the semester showed that none of them had any prior experience with PDB or PyMOL.

The activities were divided into three 2 h class sessions.

- *Session 1*. The students were instructed in the basic skills of PDB and PyMOL software required to visualize and manipulate macromolecular structures. As an example for training, the spike protein of SARS-CoV-2 was employed.
- *Session 2*. PyMOL was used to manipulate and explain the structure of the ACE2 receptor cell, along with its complexes with the SARS-CoV-2 spike protein.
- *Session 3*. Structural analysis of several antibodies in complex with the spike protein were studied. Finally, students were able to answer the question: *how is it explained chemically that vaccines save lives?*

At the end of this session, an anonymous survey was carried out in which the students were requested to assess different aspects of their experience.

### 2.1 Session 1: Learning What PDB Is, How PyMOL Software Works, and the Structural Features of the SARS-CoV-2 Spike Protein

**Figure 1:**
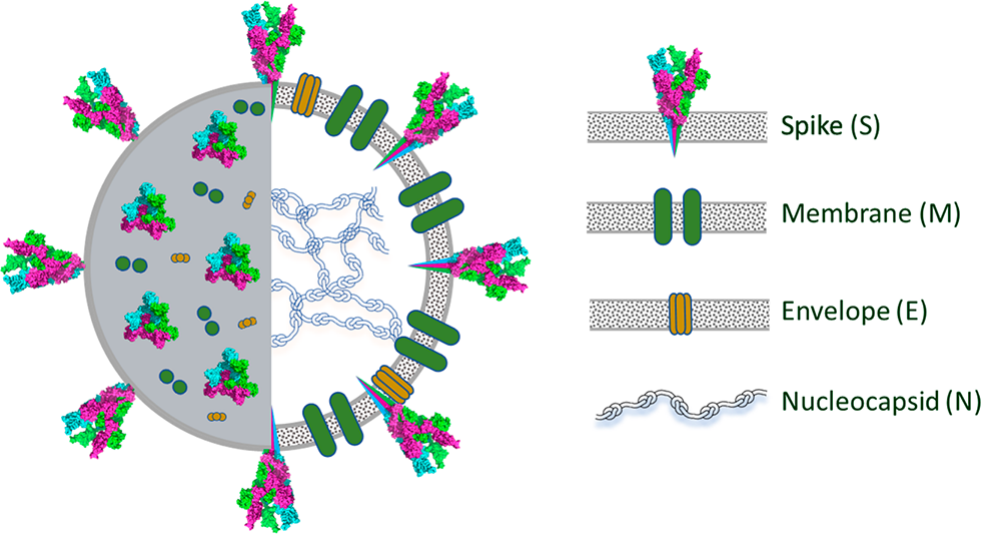
Schematic structure of the SARS-CoV-2 virion.

The instructor provided to the students a brief introduction to the most important features of the structure of SARS-CoV-2. The four major structural proteins are displayed: the envelope (E), membrane (M), nucleocapsid (N), and spike (S) proteins (Figure 1).^7^

**Figure 2:**
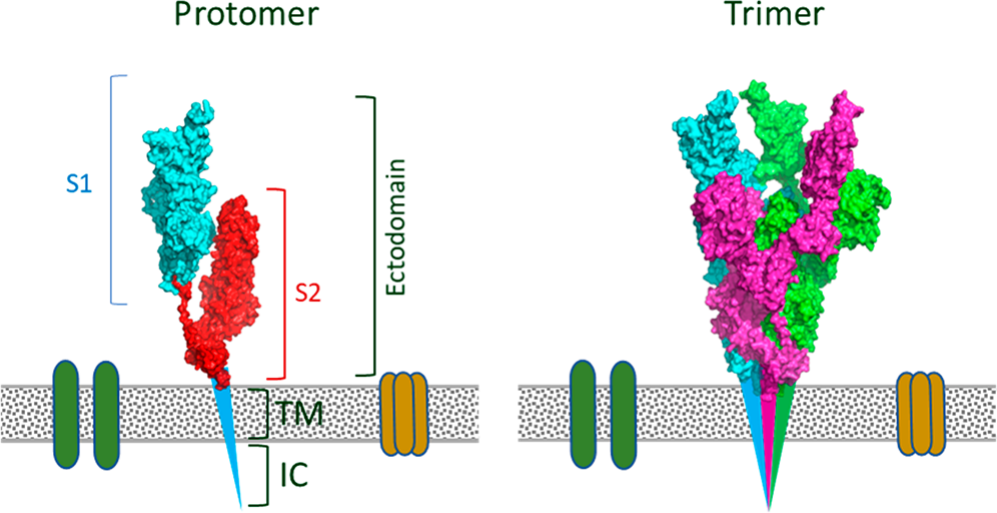
Schematic structure of the S protein protomer.

It is highlighted that spike protein (approximately 180–200 kDa) is the surface glycoprotein anchored to the viral membrane that plays an essential role when the infection process of SARS-CoV-2 takes place. This protein is a trimer of three identical protomers (Figure 2). Each protomer contains three segments: a short intracellular tail (IC), a transmembrane anchor (TM), and a large ectodomain that extends outward from the virus which is coated with sugar chains to hide the virus from the immune system^8^ and comprises S1 and S2 subunits.

Next, the students are invited to study the ectodomain by analyzing the requested structural features that they must observe manipulating PyMOL.

Although hundreds of structures of this spike protein are already available in the Protein Data Bank, the one with the code 7DWY(9) has been selected and must be loaded in a PyMOL session. They are encouraged to distinguish the four different levels of the protein structures: primary, secondary, tertiary, and quaternary, changing the representation of the molecule from lines or wireframe to cartoon.

They must learn how to select individual residues or different chains, how to change their colors, how to generate objects, how to show and hide different parts of the protein, how to measure distances and angles for bonds, and how to generate surfaces.

**Figure 3:**
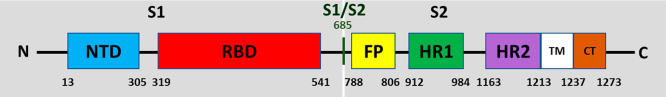
Schematic of SARS-CoV-2 spike protein primary structure. Different domains are shown by different colors. **NTD**, N-terminal domain; **RBD**, receptor-binding domain; **FP**, fusion peptide; **HR1** and **HR2**, heptad region 1 and 2; **TM**, transmembrane domain; **IC**, intracellular tail.

They have to realize that the spike protein is a complex of three identical chains. A schematic illustration of the spike protein (Figure 3) is given to the students, and they must recognize every single domain in the ectodomain, extracting them as different objects and coloring them in the suggested color.

The S1 subunit has an N-terminal domain (NTD) and a receptor-binding domain (RBD) located in the C-terminal domain, which is implied in recognition and binding to the host cell receptor. S2 consists of the fusion peptide (FP), two heptad repeats 1 (HR1 and HR2) which operate the fusion of viral and host membranes, a transmembrane domain (TM), and a cytoplasmic tail (CT).

When different species of coronavirus are compared, the S2 subunit is highly conserved, but the sequence of the S1 subunit varies greatly.

S1 and S2 are connected to the S1/S2 cleavage site in which specific proteases act. The cleavage transforms the spike protein into a fusion competent form that suffers several conformational changes and allows it to anchor to the host membrane leading to the membrane fusion.^10^

### 2.2 Session 2: Structural Features of the Angiotensin-Converting Enzyme 2 (ACE2) and Conformations of the Spike RBD Domains

**Figure 4:**
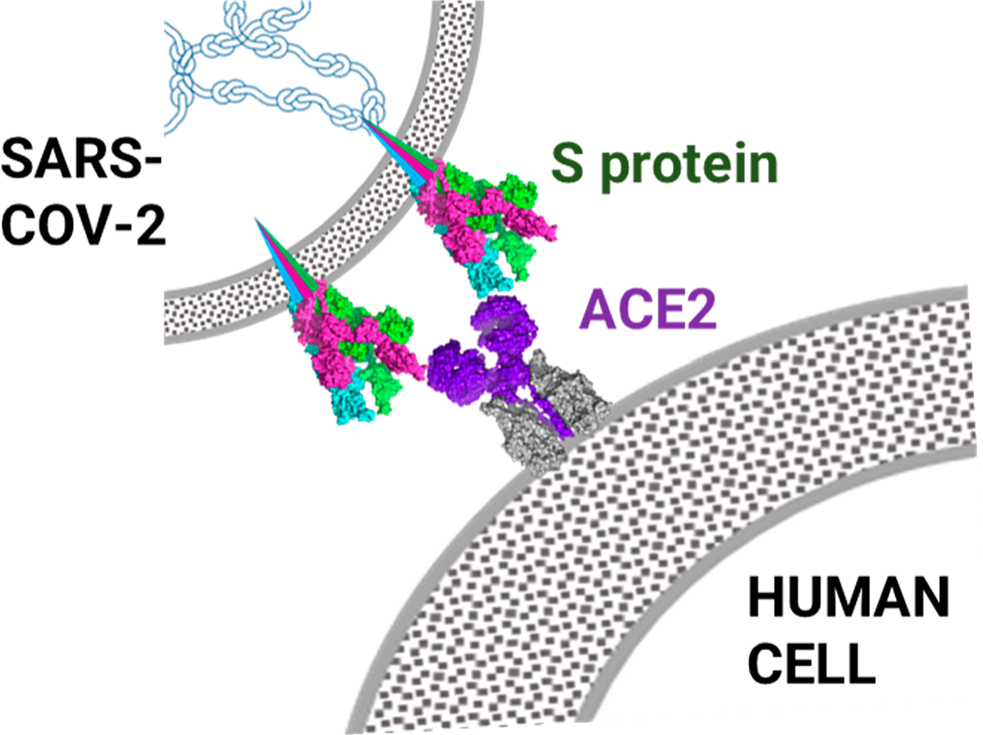
Schematic RBD-ACE2 interaction.

The instructor explains the important role of the receptor-binding domains (RBD) responsible for recognition and binding to the host cell receptors. These receptors allow binding to angiotensin-converting enzyme 2 (ACE2) that is a transmembrane protein that activates angiotensin, a peptide hormone involved in the control of blood pressure. It was discovered that ACE2 is a functional receptor for the coronavirus responsible for severe acute respiratory syndrome (SARS),^11^ and it is found on the membrane of the lung, heart, kidney, and intestinal cells, which are the perfect targets for the infection by the virus. Hence, ACE2 behaves as a cellular entrance, and the virus binds to it like a key being inserted into a lock (Figure 4).

**Figure 5:**
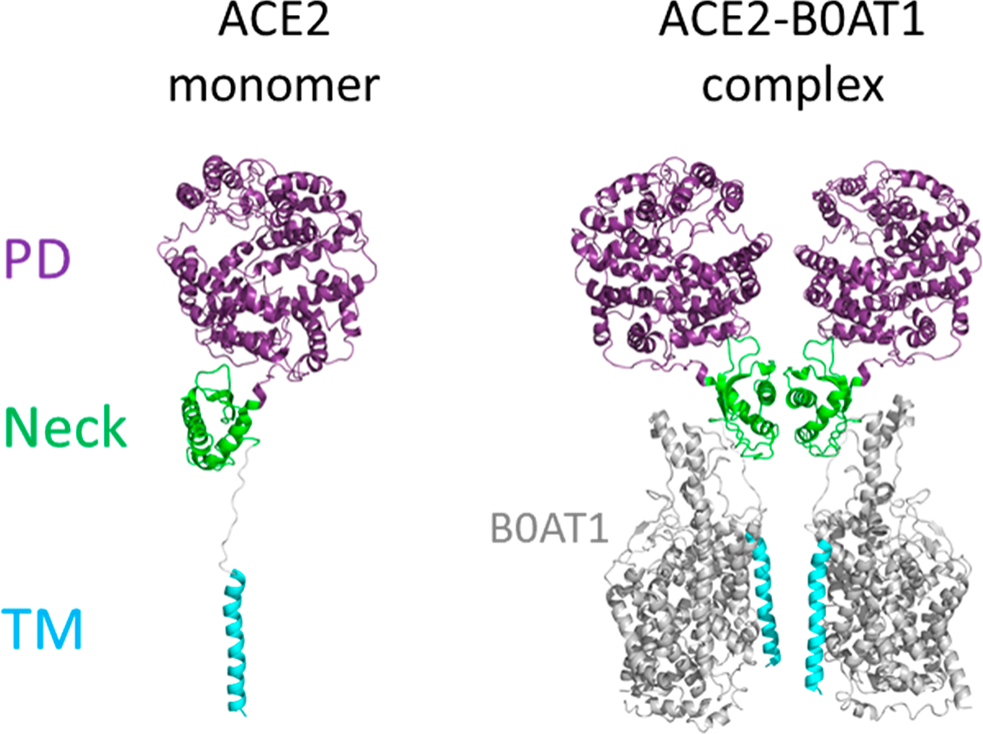
Left: monomer ACE2 domains. Right: ACE2-BOAT1 complex.

ACE2 is a homodimer with an extracellular domain and a small transmembrane domain:^12^ the catalytic peptidase domain (PD, residues from 19 to 615), the smaller neck domain (residues from 616 to 726), and the single-helix transmembrane (TM) domain (residues from 741 to 774). (See Figure 5.)

The great flexibility of the TM helix made it impossible to determine the structure of the entire protein. However, since ACE2 also acts as the chaperone for membrane-bound amino acid transporter B0AT1, the structure of full-length ACE2 could be revealed by stabilization by B0AT1. The ACE2–B0AT1 complex (ID 6M1D(13)) was isolated as a dimer of heterodimers, but other additional data support that ACE2 is a homodimer even when is not bonded to B0AT1. Each dimeric ACE2 can bind with two S protein trimers.

Students must load this complex in PyMOL and identify both monomers of ACE2 and B0AT1 and the three domains in the ACE2 chains.

On the other hand, the RBD domains of the spike protein are quite flexible, and they can adopt two distinct conformations: “up” and “down”. One, two, or three domains can be upward, but the “up” conformation is required to bind to receptors. This bending ability gives the virus its great infection capacity. Researchers have postulated that the most virulent SARS variants have more flexible RBD units, unlike the coronaviruses responsible for the ordinary cold, which are less aggressive because their RBD conformational motions are more hindered.

**Figure 6:**
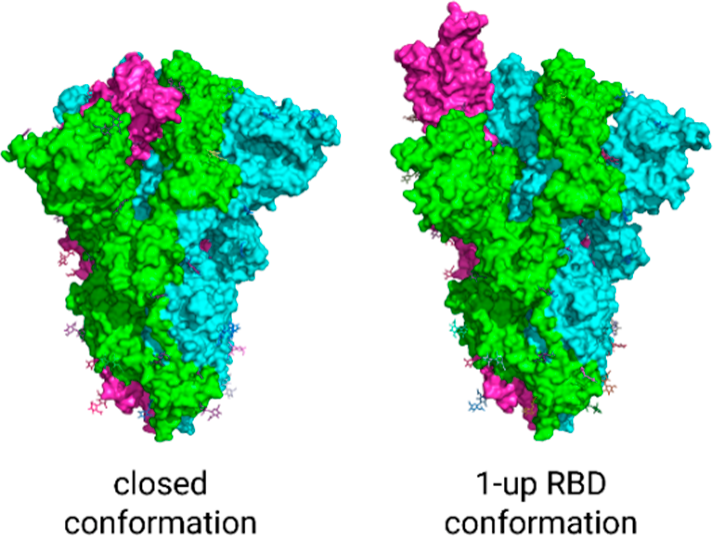
Spike protein in closed (left) and 1-up RBD (right) conformation.

In the PyMOL session named PyMOL-Session2.pse the students can now overlay the structures having PDB IDs 7DWY and 7DWZ,^9^ which present the spike in the closed and 1-up RBD conformation, respectively. The best way to visualize the different conformation of this single RBD domain is by displaying each chain of the spike protein in a different color (picking the C button in the panel and selecting “by chain” under “by chain”) and showing both proteins in the surface mode (Figure 6).

The students must compare both molecules and discuss the structural differences between them until concluding that one RBD domain is down or up according to the structure.

Next, they will turn on the objects called RBD-2-up and RBD-3-up, extracted from structures 7DX8(9) and 7DX9,^9^ respectively, in which the second and the third RBD domains can also be observed in the up conformation (Figure S1).

Next, students simultaneously turn on structures 7DWZ and 6M1D (Figure S2). They must be able to explain what they observe, that is, how the RBD domain of the spike protein interacts with the ACE2 receptor. Next, the structure with ID 7DX7(9) is turned on, and the students are asked to describe this new complex, to conclude that is the S protein of SARS-CoV-2 bound with PD of ACE2 in the called *conformation 1* (1 up RBD and 1 PD bound). They consecutively turned on structures 7DX8 and 7V8A,^14^ to characterize by themselves *conformation 2* (2 up RBD and 2 PD bound) and *conformation 3* (3 up RBD and 3 PD bound), respectively, for the same complex (Figure S3).

As the last exercise, the students must load the complex with ID 7DWX,^9^ analyze the structural features, and describe what they are visualizing as the structure of an S-ACE2-B0AT1 ternary complex, in which one ACE2 dimer binds two trimeric S proteins simultaneously (Figure S4).

Ideally, students should know how to work with PyMOL, but to make the job easier the PyMOL-Session2.pse session is provided for session 2.

### 2.3 Session 3: Answering the Question: Why Do SARS-CoV-2 Vaccines Prevent Serious Illness and Save Hundreds of Thousands of Lives?

Activity 3 starts with the instructor explaining that, after the interaction of the spike protein with the entry receptor ACE2, cleavage of the S1 domain is achieved by a protease. Proteolytic cleavage is followed by conformational changes in S2, which allows the fusion of the virus with the cellular membranes leading to the cytoplasmatic release of the viral genome into the host cell.^15^ Because the viral genome must access the cytoplasm, every step of this process is important. Understanding the foundations of these entry mechanisms allows researchers to design vaccines, antibodies, small molecule inhibitors, and other potential therapeutics targeting to prevent SARS-CoV-2 access into the host cell.

A brief outline should be also provided to students about how the body fights illness and how vaccines work. So, they must know that after bacteria or viruses enter the human body they start to multiply, giving rise to infection and causing disease. Immediately, the immune system is activated and produces antibodies to fight off the infection, but this process requires a few days, which is why we have symptoms such as fever, headache, fatigue, or body aches. After the first infection, the immune system will recognize the germ and will already know how to defend the body. Vaccines contain attenuated or inactivated parts of a specific organism which provoke a mimicked infection in the body helping the immune system to create the specific antibodies. Of course, this simulated infection can cause some symptoms which are common while the body creates the new antibodies. Vaccines are the safest and most effective way of protecting people from infections. Of course, they are not perfect and a person can develop disease despite having been vaccinated, although they will be at a much lower risk of becoming seriously ill.

Next, students load and overlay the structures with IDs: 7V2A,^16^7TB8,^17^7WPD,^18^7CZP,^19^7CZQ,^19^ and 7JZL(20) (Figure S5).

All are complexes of the spike protein with antibodies or inhibitors bonded to the receptor binding domain (RBD). They must answer the following two questions: (1) *why do SARS-CoV-2 vaccines prevent serious illness and save hundreds of thousands of lives?* And based on what they have learned: (2) *what could be the influence of virus variants on the efficacy of these antibodies, and why?*

At the end of these activities, most of the students made the connection between the observed structural features and the efficacy of vaccines, concluding by themselves that antibodies or inhibitors act by blocking the ACE2 binding of the spike protein and, as consequence, the viral entry into the host cells.

During the sessions, the students explained to the instructors their respective answers to the questions and the instructors evaluated them. In addition, a quick assessment of the student’s learning can be done using a short questionnaire as such the one provided in the SI. If desired, it can be carried out with Kahoot or similar tools.

## 3 Results and Discussion

The Bioinorganic Chemistry course is a one-semester program offered to final-year graduation students in Chemistry and Chemistry & Material Sciences at the Chemistry Faculty of Sevilla University.

The PyMOL class activities described herein have been carried out during the 2021/2022 second semester by 60 students separated into two laboratory sections. They were students of a Bioinorganic Chemistry course at a fourth-year university level from Chemistry and Chemistry & Material Sciences areas.

**Figure 7:**
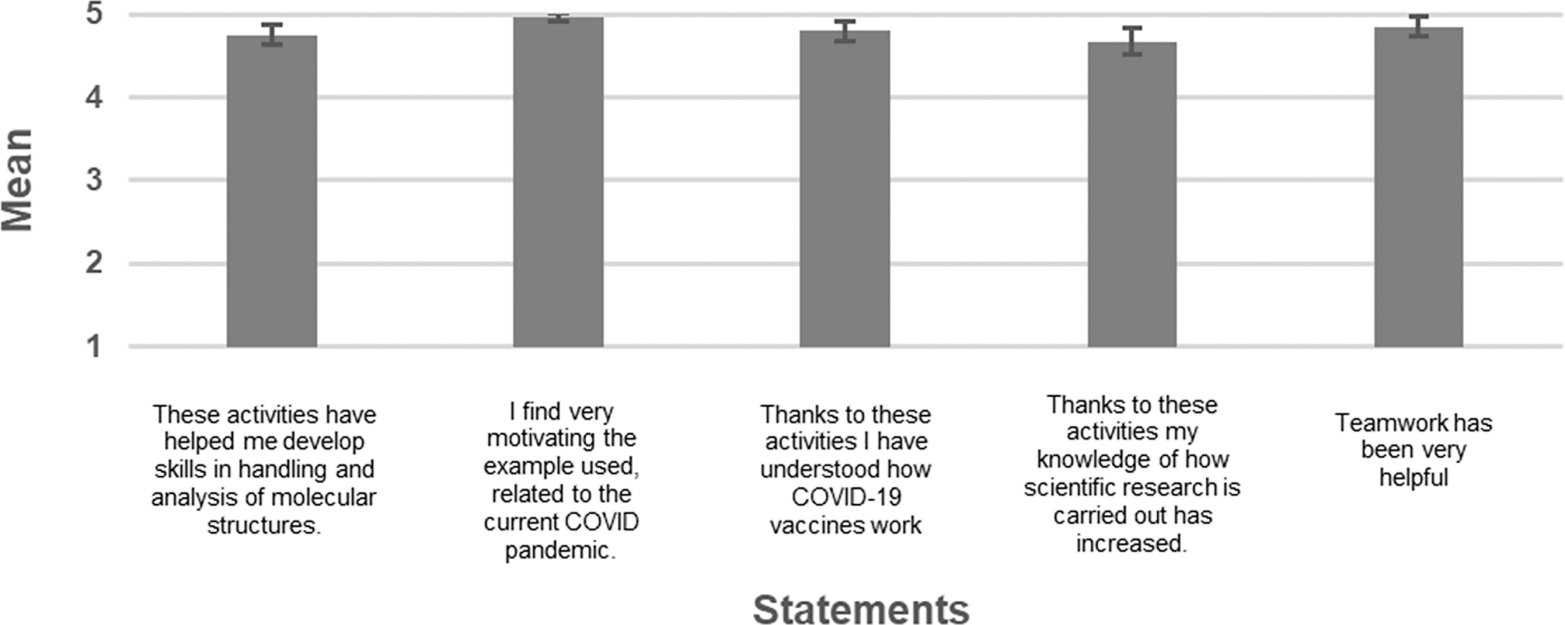


After doing the proposed activities, the students completed a survey in which they stated their level of agreement to 20 given statements. Figure 7 shows the obtained results in 5 of these statements. A 5-point Likert scale was used (1 = Fully disagree, 5 = Fully Agree). The students answered the survey anonymously using a provided Google form which provides all the responses automatically.

Based on this assessment, students judged these exercises help them develop their skills in handling and analysis of molecular structures (95.0%), overwhelmingly found the activity very interesting and motivating (99.4%), and helpful in understanding the impact of vaccination campaigns on reducing the incidence, hospitalizations, and deaths for COVID-19 (96.0%).

In addition, students feel that they have learned how scientific knowledge is generated and shared (93.4%).

Their enthusiasm can be also noticed in the students’ notes. Some students commented in front of the entire class:

“I find very motivating that the example used in this practice is related to the current COVID pandemic because it allows me to understand how infection takes place and what vaccines are for.”

“Now I can understand why COVID-19 vaccines prevent serious illness and death helping to stop the global pandemic.”

Students highly appreciated the efforts of their teachers who conducted the activity for them and considered the teamwork very helpful (97.0%).

When asked how to implement improvements students said that they would like to learn in a similar way about other viruses and diseases.

## 4 Conclusions

A series of engaging exercises are described in which students emulate the process that researchers have used to efficiently develop COVID-19 vaccines or rational drug design.

Thanks to these activities, students can understand that the S protein plays a key role in the infection process of SARS-CoV-2 to human cells. They learn that the S protein contains three receptor-binding domains (RBD) which allow binding to the peptidase domain (PD) of the angiotensin-converting enzyme 2 (ACE2), a protein on the surface of many cell types. Hence, ACE2 acts as a cellular doorway—a receptor—and the virus binds to it like a key being inserted into a lock.

While they discover about protein structure and protein–ligand interactions using the PyMOL software, through the process, the students also learn about infectious processes, computational drug design, and how scientific knowledge is constructed.

Based on our assessment, students enjoy the exercises, understand the importance of the structural analysis of biomolecules, become more interested in science research, and demonstrate increased knowledge of content relevant to the topics.

## Supporting Information Available

Session 1 instruction sheet (PDF)
Session 2 instruction sheet (PDF)
Session 3 instruction sheet (PDF)
PDB IDs table (PDF)
Figures S1–S5 (PDF, DOCX)
Session 1 lab report (PDF, DOCX)
Session 2 lab report (PDF, DOCX)
Session 3 lab report (PDF, DOCX)
Test/assessment (PDF, DOCX)
PyMOL-Session2 (ZIP)
