# Supplementary material for: Using PyMOL to Understand Why COVID-19 Vaccines Save Lives
Source: J Chem Educ. 2023 Feb 28;100(3):1351–6. doi: 10.1021/acs.jchemed.2c00779 (PMC9999942; doi:10.1021/acs.jchemed.2c00779)
Supplement: Supplementary file 1 — ed2c00779_si_001.pdf [file ed2c00779_si_001.pdf]

Using PyMOL to understand why COVID-19 vaccines save lives.

Celia Maya*

Instituto de Investigaciones Químicas (IIQ), Departamento de Química Inorgánica and Centro de Innovación en Química Avanzada (ORFEO-CINQA)

Consejo Superior de Investigaciones Científicas (CSIC) and University of Seville

Avda. Américo Vespucio, 49, 41092 Sevilla (Spain)

* maya@us.es

- **Session 1**
- **INSTRUCTIONS SHEET 1**

Session 1

Activities.

Before coming to class, the students must:

- Install PyMOL in their computers.

- Find in internet images of SARS-CoV-2 and identify the Spike (S) protein on the surface.

In class:

<u>Instructor</u>: Introduction

- A brief presentation about the Protein Data Bank (PDB) as a database for the 3D structures of large biological molecules, such as proteins and nucleic acids.
- A brief presentation about PyMOL as a powerful molecular visualization system to analyse molecular structures and making publication quality pictures or movies. It is an essential tool for any molecular modeller as it allows to understand the structural features of biomolecules and their interactions. The instructor launchs PyMOL and explains that there are two windows, the Viewer Window, where the molecules appear, and the Upper Control Window (UC window). It is important to emphasize that there are two equivalent command lines at the bottom of each PyMol window.

<u>Students</u>: Worksheet 1 and Lab Report 1.

They must work following the instructions provided by the instructor in Worksheet 1.

Finally, they will write a lab report on Session 1 including all the images created during the activity, as well as the answers to the questions raised. (A detailed word document will be given to complete it.)

INSTRUCTIONS SHEET 1

1.- Start PyMOL and load the S protein structure with pdb code 7dwy.[1]

To do so: type fetch 7dwy

The molecule is now in your viewer window and an object named '7dwy' appears with light grey backgrounds in the upper right side of the Viewer window.

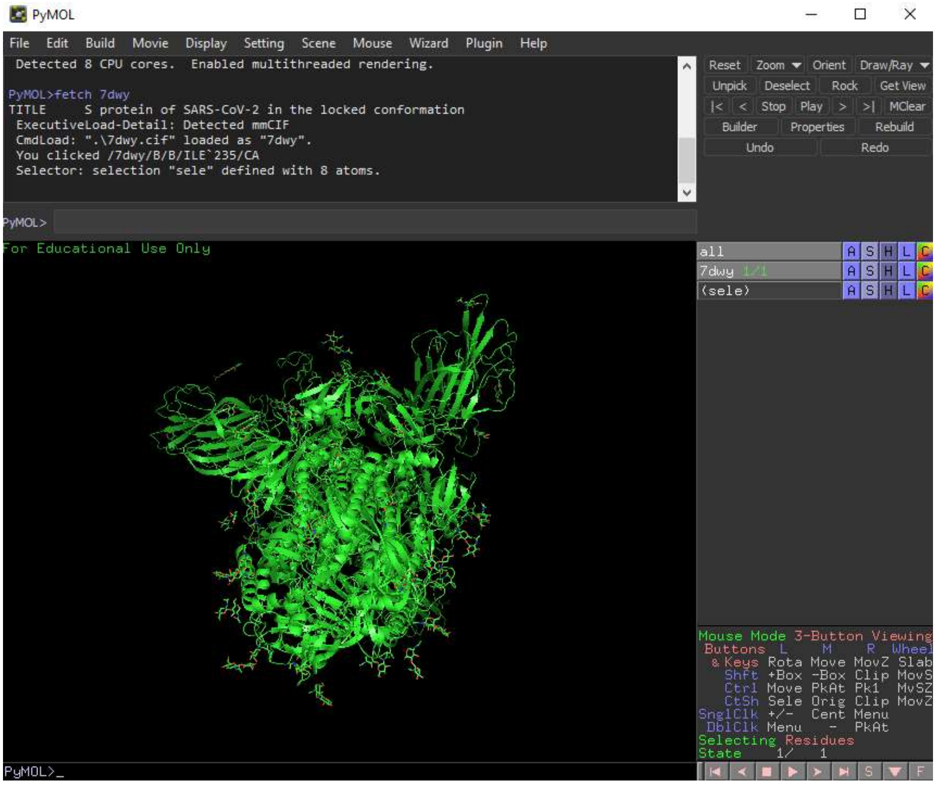

Figure 1

Click on the name to deactivate, or disable, the object, and it will be hidden from view. You will have entries for 'all' and '7dwy', and maybe 'sele'.

2.- Move your protein.

To do so: Locate your mouse cursor over the protein and:

Left mouse button rotates the molecule, *middle mouse button* moves the molecule in the plane of the display and dragging with the right mouse button zooms in and out.

[1] Yan, R., Zhang, Y., Li, Y., Ye, F., Guo, Y., Xia, L., Zhong, X., Chi, X., Zhou, Q. *Structural basis for the different states of the spike protein of SARS-CoV-2 in complex with ACE2. Cell Research* ***2021,*** 31, 717–719.

Clicking the Reset button in the UC window the original view is always restored.

There are 2 mouse modes: *Viewing Mode* (default) and *Editing Mode* to physically move atoms, residues, etc. You can cycle between the 2 modes by clicking in the Mouse Mode Table (right bottom of the Viewer Window).

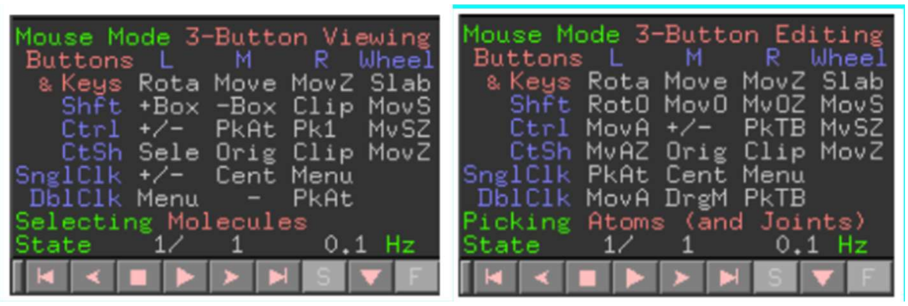

Figure 2.

Clicking over ‘Selecting’ in Viewing Mode you can decide what kind of element (Residues, Chains, Segments, Objects, Molecules, C-alphas, or individual Atoms) you want to select clicking with the mouse.

The chosen elements will be added to a special object called ‘sele’.

3.- Select a chain in the protein and colour it red.

To do so: select ‘chains’ in the Viewing Mouse Mode and click on any point of the structure. A whole chain is selected.

Next to each object you can see five letters: ASHLC. A drop-down menu is displayed when you click on each letter**.**

A = Actions → contains lots of preset actions.

S = Show → allows us to control various representations (lines, sticks, ribbon, cartoon, surface…)

H = Hide → is the opposite of Show, you can hide various representations

L = Label → for showing and creating labels

C = Color → for colouring the objects

Color the chain by choosing C→Color→Reds→Red in the object ‘sele’.

4.- Create a new object for this chain.

To do so: choose A→Action→Create Object for the selection.

A new object name will appear in the names list, and you can then rename this object using A→Actions→Rename.

5.- Show the protein as lines, cartoon, or ribbon.

To do so: hide everything in your PyMOL window by clicking H→Hide→Everything in the all object row. Selecting S→Show→Lines backbone and side chains are displayed.

Now show S→Show→Cartoon. The molecule will be shown as lines and cartoons because PyMOL's show command acts additively. Now show Ribbon. The ribbon traces the backbone of the protein.

The Hide menu works like the Show menu: when you turn off a representation, other representations are left unchanged. For example, please hide the Lines representation by choosing H→Hide→Lines. Just the ribbons and cartoons should be showing now.

6.- Show the protein as cartoon. Identify all the chains of the Spike protein. Create an object for each chain and name them as 'ChainA' (magenta), 'ChainB' (cyan) and 'ChainC' (green). Colour them in the indicated colour.

Save an image and copy it in your answers sheet. (Picture 1)

(You can learn how to save or copy images at the end of this instructions sheet).

7.- Show the protein in the surface mode and colour each chain in a different colour.

To do so: choose S→Show→As→Surface.

Save an image and copy it in your answers sheet. (Picture 2)

8.- Turn on the sequence of your protein.

To do so: click on Display→Sequence from the Upper Control Window.

Now, the sequence of the protein in the one-letter codes is displayed. The sequence viewer can be used to select residues.

Scroll to the right end of the protein sequence in the sequence viewer and you will see a few residues named with 3-letter codes that are not amino acids. For example, NAG is the code for the ligand N-acetyl-D-glucosamine. (an amide derivative of the monosaccharide glucose, part of a biopolymer in the bacterial cell wall).

Water molecules are written as a row of O's at the end of the sequence.

9.- Using the protein represented in the Surface mode, select the residues from V16 to F676 in ChainA, ChainB and ChainC. Create 3 objects and name them 'S1-A', 'S1-B' and 'S1-C', respectively.

(Don't forget to select 'Residues' in the Viewing Mouse Mode.)

Save an image with only these 3 objects and copy it in your answers sheet. (Picture 3)

10.- Using the protein represented in the Surface mode, select the residues from Q690 to D1146 in ChainA, ChainB and ChainC. Create 3 objects and name them 'S2-A', 'S2-B' and 'S2-C', respectively.

Save an image with only these 3 objects and copy it in your answers sheet. (Picture 4)

11.- Show ChainA in Sticks mode. Select the residues C15, V16, N17 y C136. Create an object with this selection colouring by element. Name it as 'Measure'. Label each atom with its element symbol. Locate this element in the center of the screen (To do so: A→orient**)**

Save an image with only this object and copy it in your answers sheet. (Picture 5)

12.- Using the object 'Measure', take measurements of bond distances, angles, and dihedral angles in residue C15.

To do so: click on Wizard→Measurement. PyMOL provide controls for the user on the right side above the Mouse Mode matrix (Figure 3).

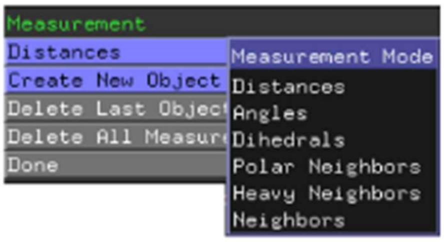

Figure 3.

Next, click on any two atoms; the distance between the two should now be annotated by a dashed line and distance measure.

To change the type of measurement, click on the top button in the measurement wizard (Distances). A menu will pop up, giving you the other options. If you want to measure Angles, you need to select three atoms. Dihedrals require four atoms. Polar, Heavy and all Neighbors need just one.

By default, each measurement creates a new object. You can change this by merging the new object with the previous one, or by overwriting the previous object.

Don't forget, when you're done using a Wizard, make sure you click Done—the last button in the wizard.

Complete Picture 6 with measurements in your answers sheet.

13.- Using the object 'Measure', take measurements of the S-S bond distance and explain what this bond is. Are there any more disulfides bonds in a Spike protomer.

Write answers in your answers sheet.

14.- Create an object for every single domain in a Spike protomer, colouring them in the suggested colour.

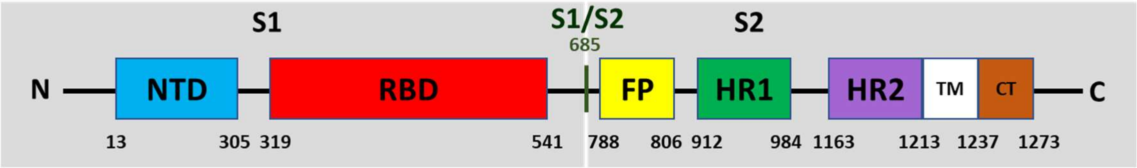

Figure 4.
Schematic structure S protein. **NTD**, N-terminal domain; **RBD**, receptor-binding domain; **FP**, fusion peptide; **HR1** and **HR2**, heptad region 1 and 2; **TM**, transmembrane domain; **IC**, intracellular tail.

Save an image in your answers sheet. (Picture 7)

<u>Save images:</u>

Once your molecule is set up, click on 'Draw/Ray' in the upper right side of the Upper Control Window (Figure 5).

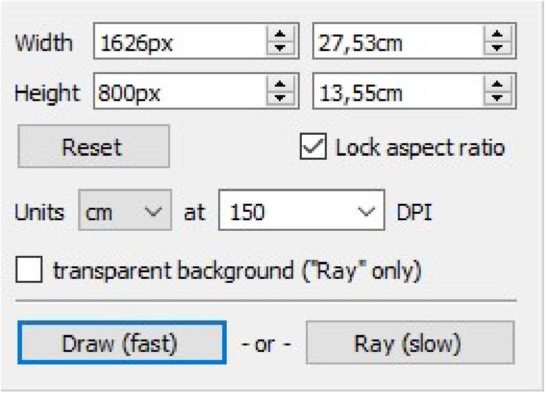

Figure 5

Images for presentation or display on a computer can be set to default, 150 DPI. Images for printing or for publication are required to be 400 DPI or higher.

You can fast save images choosing the Draw option. However, if you want to save them with superior detail press the Ray button. Ray tracing smoothes edges, adds shadows and generates better lighting effects, although takes much longer. To save a high resolution image you need to fix the size of image.

Another property that is useful to know about is transparency. If an image has a transparent area, then whatever is underneath the image will show through. By default, the image has an opaque background. If you want a transparent one you must check the box before click on Ray button. Lastly, a new window is open and you can save image as png or copy the image to the clipboard.
