# Supplementary material for: Using PyMOL to Understand Why COVID-19 Vaccines Save Lives
Source: J Chem Educ. 2023 Feb 28;100(3):1351–6. doi: 10.1021/acs.jchemed.2c00779 (PMC9999942; doi:10.1021/acs.jchemed.2c00779)
Supplement: Supplementary file 2 — ed2c00779_si_002.pdf [file ed2c00779_si_002.pdf]

Using PyMOL to understand why COVID-19 vaccines save lives.

Celia Maya*

Instituto de Investigaciones Químicas (IIQ), Departamento de Química Inorgánica and Centro de Innovación en Química Avanzada (ORFEO-CINQA)

Consejo Superior de Investigaciones Científicas (CSIC) and University of Seville

Avda. Américo Vespucio, 49, 41092 Sevilla (Spain)

* maya@us.es

- **Session 2**
- **INSTRUCTIONS SHEET 2**

Session 2

Activities.

Before coming to class, the students must:

- Send the Lab Report of Session 1.

In class:

Instructor: Introduction

- A brief presentation about the important role of the receptor-binding domains (RBD) of the Spike proteins and their two conformations ('up' and 'down').
- An overview of the structure and function of the angiotensin-converting enzyme 2 (ACE2).

Students: Worksheet 2 and Lab Report 2.

They must work following the instructions provided by the instructor in Worksheet 2.

Finally, they will write a lab report on Session 2 including all the images created during the activity, as well as the answers to the questions raised. (A detailed word document will be given to complete it.)

Note for instructors:

Instructions 1, 4, 5, 6, 8, 9, 10, 11, 12, 16, 18, and 21 can be skipped, using instead the provided PyMOL session (PyMOL-Session2.pse)

INSTRUCTIONS SHEET 2

1.- Load the ACE2-B0AT1 complex with pdb code 6m1d.[1]

To do so: type fetch 6m1d

2.- Identify the two monomers of ACE2 and B0AT1 in 6m1d. Colour grey both monomers of B0AT1.

3.- Show in the indicated colour each domain of the ACE2 monomers:

- **the peptidase domain (PD, residues 5–615): violet**
- **the neck domain (ND, residues 616–730): green**
- **the transmembrane domain ((TM, residues 731-774): cyan**

Save an image and copy it in your answers sheet. (Picture 1)

4.- Load the structures with pdb codes: 7dwy[2] **and 7dwz**[2]**.**

5.- Align 7dwz with 7dwy.

To do so: type align 7dwz, 7dwy

6.- Show both proteins in the surface mode displaying each chain in a different colour

To do so:

- choose S→Show→As→Surface
- picking C button in the panel and select 'by chain' under 'by chain'.

7. Compare the structures 7dwy and 7dwz and explain differences between them.

Save an image of both superimposed structures and copy it in your answers sheet. (Picture 2)

Write your explanation.

8.- Load the structures with pdb codes: 7dx8[2] **and 7dx9**[2]**.**

9.- Align 7dx8 and 7dx9 with 7dwy.

10.- For structure 7dx8, create an object with only the spike protein.

To do so:

- select the 3 chains using 'chains' in the Viewing Mouse Mode.
- choose A→Action→Create Object for the selection

---

[1] Renhong, Y., Yuanyuan, Z., Yaning, L., Xia, L., Yingying, G., Qiang, Z. Structural basis for the recognition of SARS-CoV-2 by full-length human ACE2. Science ***2020,*** *367*(6485), 1444-1448.

[2] Renhong, Y., Yuanyuan, Z., Yaning, L., Ye, F., Guo, Y., Xia, L., Zhong, X., Chi, X., Zhou, Q. *Structural basis for the different states of the spike protein of SARS-CoV-2 in complex with ACE2. Cell Research* ***2021,*** *31*, 717–719.

- rename the new object as **RBD-2-up**.

11.- For structure 7dx9, repeat step 6, naming the object as RBD-3-up.

12.- Show objects in the surface mode displaying each chain in a different colour.

13.- Activate the structures 7dwy and 7dwz and the objects RBD-2-up and RBD-3-up.

14. Compare structures and objects and explain differences between them.

Save an image of all superimposed structures and copy it in answers sheet. (Picture 3)

Write your explanation.

15.- Deactivate all objects and align 6m1d with 7dx9.

16.- Activate the structures 6m1d and 7dx9.

17.- Explain what you observe.

Write your explanation.

18.- Load the structures with pdb codes: 7dx7[2] and 7v8a[3] and align both with 7dwy. Show them in the surface mode displaying each chain in a different colour.

19.- Activate consecutively the structures 7dwy, 7dx7, 7dx8 and 7v8a.

20.- Compare these structures and explain differences between them.

Save an image of each structure and copy them in your answers sheet. (Pictures 4-7)

Write your explanation.

21.- Load the complex with pdb code: 7dwx,[2] align with 7dwy and show it in the surface mode and display each chain in a different colour.

22.- Analyse the structural features of structure 7dwx and describe what you are visualizing.

Save an image of structure and copy it in your answers sheet. (Picture 8)

Write your description.

---

[3] Yang, T.J., Yu, P.Y., Chang, Y.C., Hsu, S.T.D. Cryo-EM structure of SARS-CoV-2 S-Delta variant (B.1.617.2) in complex with Angiotensin-converting enzyme 2 (ACE2) ectodomain, three ACE2-bound form conformation 2. To be published. Deposited on PDB in 2021-08-22.
