# Supplementary material for: Using PyMOL to Understand Why COVID-19 Vaccines Save Lives
Source: J Chem Educ. 2023 Feb 28;100(3):1351–6. doi: 10.1021/acs.jchemed.2c00779 (PMC9999942; doi:10.1021/acs.jchemed.2c00779)
Supplement: Supplementary file 3 — ed2c00779_si_003.pdf [file ed2c00779_si_003.pdf]

Supporting Information

Using PyMOL to understand why COVID-19 vaccines save lives.

Celia Maya*

Instituto de Investigaciones Químicas (IIQ), Departamento de Química Inorgánica and Centro de Innovación en Química Avanzada (ORFEO-CINQA)

Consejo Superior de Investigaciones Científicas (CSIC) and University of Seville

Avda. Américo Vespucio, 49, 41092 Sevilla (Spain)

* maya@us.es

- **Session 3**
- **INSTRUCTIONS SHEET 3**

Session 3

Activities.

Before coming to class, the students must:

- Send the Lab Report of Session 2.

In class:

Instructor: Introduction

- Explanation about how the spikes bind to human cells via the ACE2 receptor and then dramatically change shape driving the membrane fusion.
- Useful videos from Youtube can be used, as for example:

https://www.youtube.com/watch?v=Xuc9D4LVJdg

https://youtu.be/e2Qi-hAXdJo

Students: Worksheet 3 and Lab Report 3.

They must work following the instructions provided by the instructor in Worksheet 3.

Finally, they will write a lab report on Session 3 including the answers to the questions raised. (A detailed word document will be given to complete it.)

INSTRUCTIONS SHEET 3

1.- Load the structures with pdb codes: 7v2a,[1] 7tb8,[2] 7wpd,[3] 7czp,[4] 7czq,[4] and 7jzl.[5]

To do so: type fetch code

2.- Align all structures with 7v2a.

3.- Search these structures in the Protein Data Bank and find out what these structures are.

Write findings in your answers sheet.

4.- Answer the following question:

Why vaccines prevent SARS-CoV-2 infections and save hundreds of thousands of lives?

Write answer in your answers sheet.

---

[1] Liu, Z., Xu, W., Chen, Z., Fu, W., Zhan, W., Gao, Y., Zhou, J., Zhou, Y., Wu, J., Wang, Q., Zhang, X., Hao, A., Wu, W., Zhang, Q., Li, Y., Fan, K., Chen, R., Jiang, Q., Mayer, C.T., Schoofs, T., Xie, Y., Jiang, S., Wen, Y., Yuan, Z., Wang, K., Lu, L., Sun, L., Wang, Q. An ultrapotent pan-beta-coronavirus lineage B (beta-CoV-B) neutralizing antibody locks the receptor-binding domain in closed conformation by targeting its conserved epitope. *Protein & Cell* ***2022,****13*, 655–675.

[2] To be published Preprint in BioRxiv: https://www.biorxiv.org/content/10.1101/2021.12.27.474307v1 Zhou, T. Deposited on PDB: 2021-12-21

[3] Yin, W., Xu, Y., Xu, P., Cao, X., Wu, C., Gu, C., He, X., Wang, X., Huang, S., Yuan, Q., Wu, K., Hu, W., Huang, Z., Liu, J., Wang, Z., Jia, F., Xia, K., Liu, P., Wang, X., Song, B., Zheng, J., Jiang, H., Cheng, X., Jiang, Y., Deng, S.J., Xu, H.E. Structures of the Omicron spike trimer with ACE2 and an anti-Omicron antibody. *Science* ***2022,*** 375, 1048-1053.

[4] Yan, R., Wang, R., Ju, B., Yu, J., Zhang, Y., Liu, N., Wang, J., Zhang, Q., Chen, P., Zhou, B., Li, Y., Shen, Y., Zhang, S., Tian, L., Guo, Y., Xia, L., Zhong, X., Cheng, L., Ge, X., Zhao, J., Wang, H.W., Wang, X., Zhang, Z., Zhang, L., Zhou, Q. Structural basis for bivalent binding and inhibition of SARS-CoV-2 infection by human potent neutralizing antibodies. *Cell Res* ***2021,*** *31*, 517-525.

[5] Cao, L., Goreshnik, I., Coventry, B., Case, J.B., Miller, L., Kozodoy, L., Chen, R.E., Carter, L., Walls, A.C., Park, Y.J., Strauch, E.M., Stewart, L., Diamond, M.S., Veesler, D., Baker, D. De novo design of picomolar SARS-CoV-2 miniprotein inhibitors. *Science* ***2020,*** *370*, 426-431.
