# Supplementary material for: Using PyMOL to Understand Why COVID-19 Vaccines Save Lives
Source: J Chem Educ. 2023 Feb 28;100(3):1351–6. doi: 10.1021/acs.jchemed.2c00779 (PMC9999942; doi:10.1021/acs.jchemed.2c00779)
Supplement: Supplementary file 4 — ed2c00779_si_004.pdf [file ed2c00779_si_004.pdf]

Supporting Information

Using PyMOL to understand why COVID-19 vaccines save lives.

Celia Maya*

Instituto de Investigaciones Químicas (IIQ), Departamento de Química Inorgánica and Centro de Innovación en Química Avanzada (ORFEO-CINQA)

Consejo Superior de Investigaciones Científicas (CSIC) and University of Seville

Avda. Américo Vespucio, 49, 41092 Sevilla (Spain)

* maya@us.es

Structures used in the activities – PDB ID

There are a lot of structures in PDB for the SARS-CoV-2 Spike protein and its complexes with other proteins, inhibitors, or antibodies. The activities included in this work were inspired by reading the article: *"Structural basis for the different states of the spike protein of SARS-CoV-2 in complex with ACE2."* R. Yan *et al* (2021) Cell Res 31: 717-719. Consequently, the structures 7DWX, 7DWY, 7DWZ, 7DX7, 7DX8, and 7DX9 have been taken from that article.

6M1D is the only assembly found with the ACE2 system not complexed with a SARS protein or fragment. It was used to present the ACE2 receptor.

Finally, the structures 7V2A, 7TB8, 7WPD, 7CZP, 7CZQ, and 7JZL have been chosen as examples of the S protein of SARS-CoV-2 bounded with antibodies or inhibitors. Other examples can be used.

Following, the title of each structure is written next to its PDB ID:

7DWY: S protein of SARS-CoV-2 in the locked conformation

7DWZ: S protein of SARS-CoV-2 in the active conformation

7DX7: Trypsin-digested S protein of SARS-CoV-2 bound with PD of ACE2 in the conformation 1 (1 up RBD and 1 PD bound)

7DX8: Trypsin-digested S protein of SARS-CoV-2 bound with PD of ACE2 in the conformation 2 (2 up RBD and 2 PD bound)

7DX9: Trypsin-digested S protein of SARS-CoV-2 bound with PD of ACE2 in the conformation 3 (3 up RBD and 2 PD bound)

7DWX: Conformation 1 of S-ACE2-B0AT1 ternary complex

6M1D: ACE2-B0AT1 complex, open conformation

7V8A: Cryo-EM structure of SARS-CoV-2 S-Delta variant (B.1.617.2) in complex with Angiotensin-converting enzyme 2 (ACE2) ectodomain, three ACE2-bound form conformation 2

7V2A: SARS-CoV-2 Spike trimer in complex with XG014 Fab (antibody)

7TB8: Cryo-EM structure of SARS-CoV-2 spike in complex with antibodies B1-182.1 and A19-61.1

7WPD: SARS-CoV-2 Omicron Variant S Trimer complexed with one JMB2002 Fab (antibody)

7CZP: S protein of SARS-CoV-2 in complex bound with P2B-1A1 (antibody)

7CZQ: S protein of SARS-CoV-2 in complex bound with P2B-1A10 (antibody)

7JZL: SARS-CoV-2 spike in complex with LCB1 (2RBDs open) (Inhibitor)
