# Supplementary material for: Using PyMOL to Understand Why COVID-19 Vaccines Save Lives
Source: J Chem Educ. 2023 Feb 28;100(3):1351–6. doi: 10.1021/acs.jchemed.2c00779 (PMC9999942; doi:10.1021/acs.jchemed.2c00779)

Supporting Information

Using PyMOL to understand why COVID-19 vaccines save lives.

Celia Maya*

Instituto de Investigaciones Químicas (IIQ), Departamento de Química Inorgánica and Centro de Innovación en Química Avanzada (ORFEO-CINQA)

Consejo Superior de Investigaciones Científicas (CSIC) and University of Seville

Avda. Américo Vespucio, 49, 41092 Sevilla (Spain)

* maya@us.es

**Figures S1-S5**

**Figure S1**


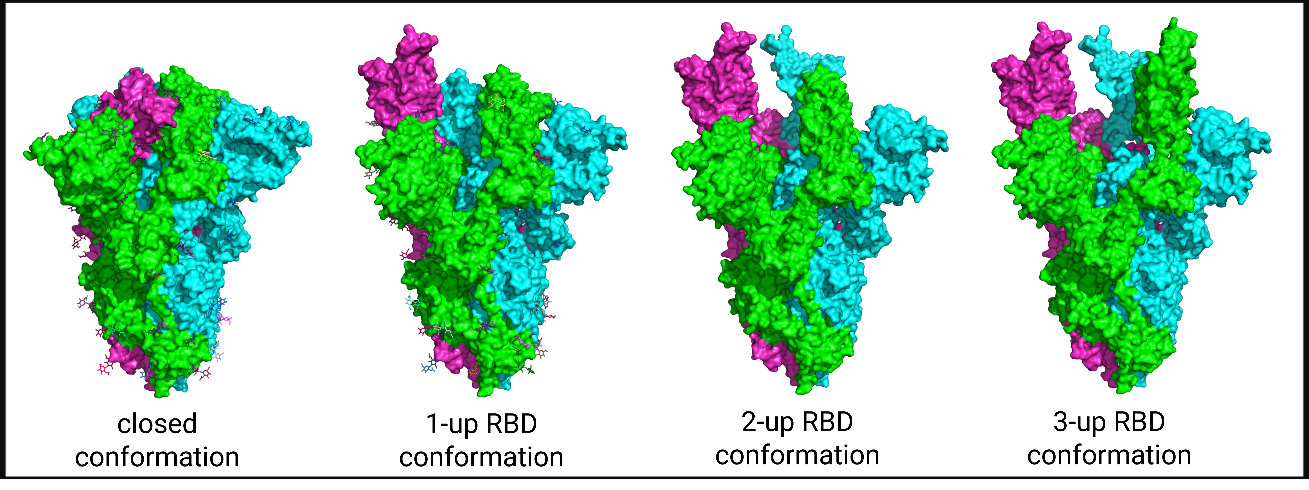


**Figure S2**


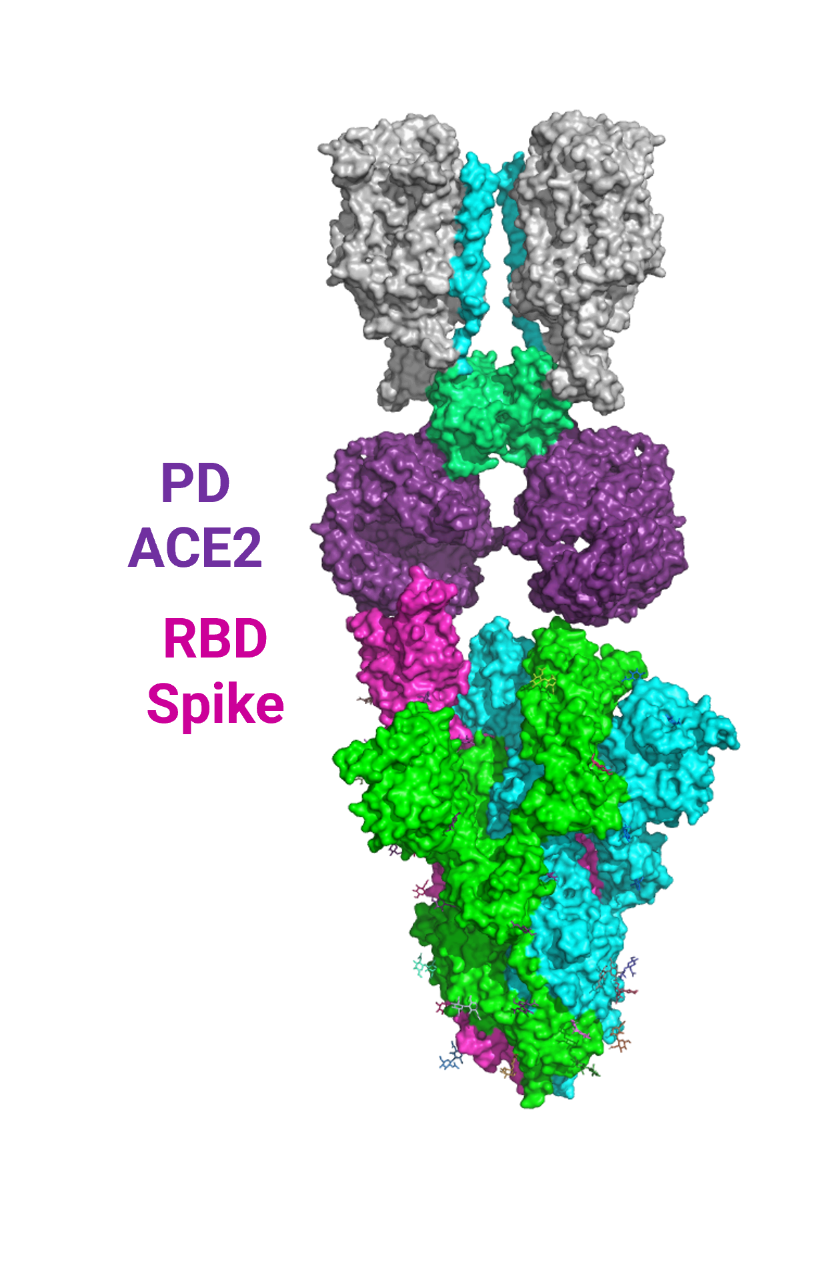


**Figure S3**


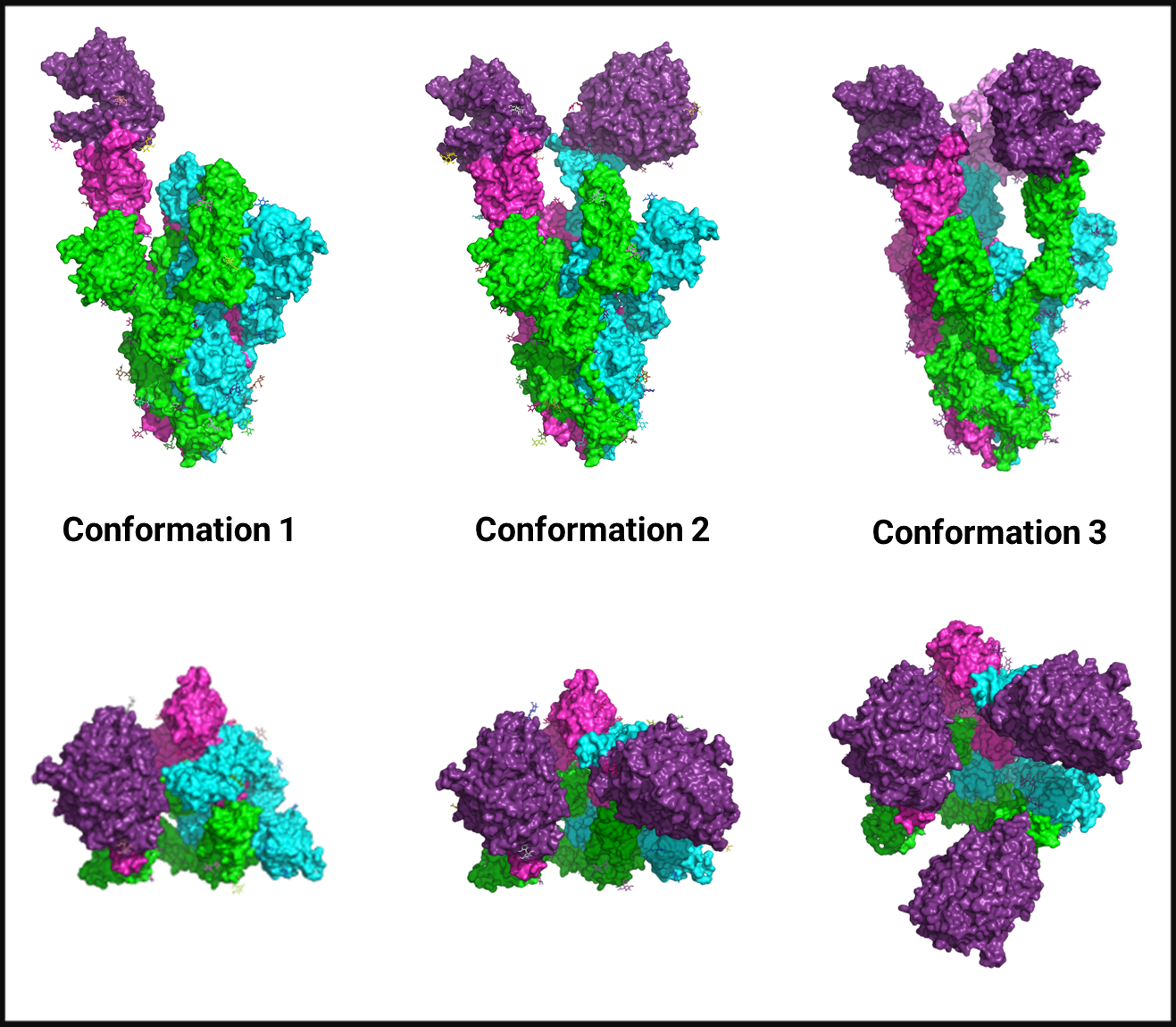


**Figure S4**


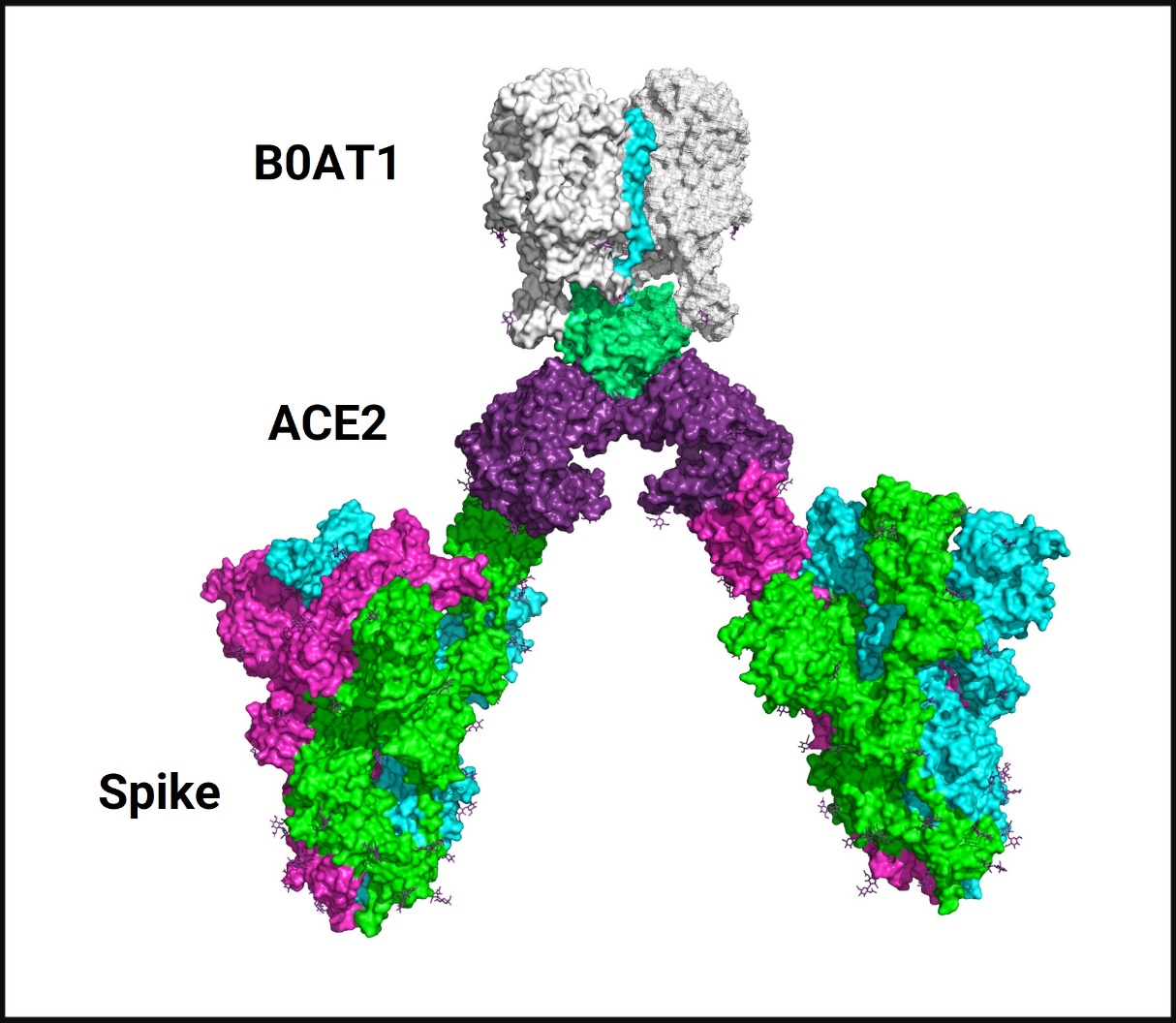


**Figure S5**


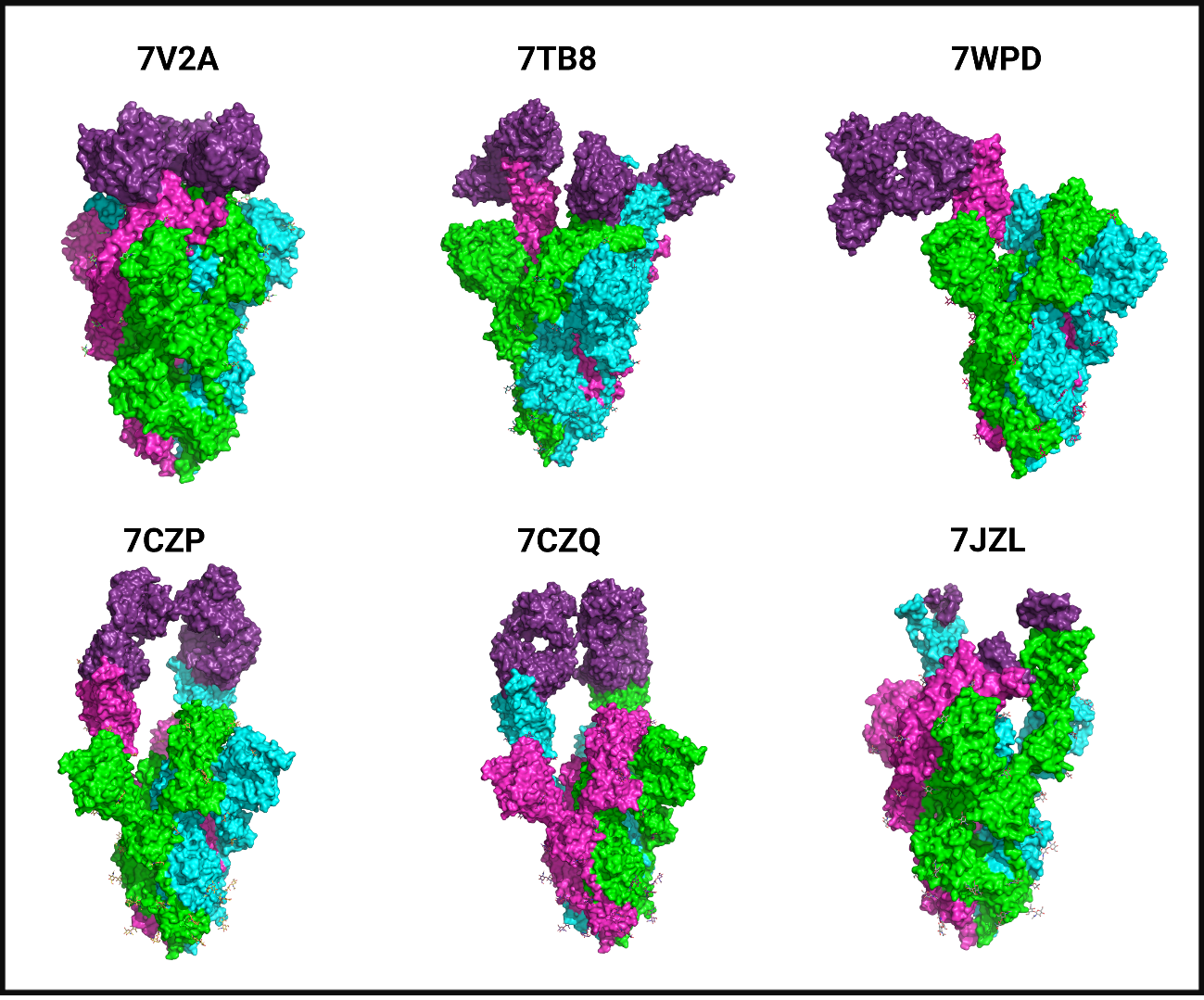

Supplement: Supplementary file 6 — ed2c00779_si_006.docx [file ed2c00779_si_006.docx]
