# Supplementary material for: Using PyMOL to Understand Why COVID-19 Vaccines Save Lives
Source: J Chem Educ. 2023 Feb 28;100(3):1351–6. doi: 10.1021/acs.jchemed.2c00779 (PMC9999942; doi:10.1021/acs.jchemed.2c00779)
Supplement: Supplementary file 10 — ed2c00779_si_010.docx [file ed2c00779_si_010.docx]

Supporting Information

Using PyMOL to understand why COVID-19 vaccines save lives.

Celia Maya*

Instituto de Investigaciones Químicas (IIQ), Departamento de Química Inorgánica and Centro de Innovación en Química Avanzada (ORFEO-CINQA)

Consejo Superior de Investigaciones Científicas (CSIC) and University of Seville

Avda. Américo Vespucio, 49, 41092 Sevilla (Spain)

* maya@us.es

- **Lab Report – Session 2**

**Lab Report – Session 2**

Insert Picture 1 *(Instruction 3)*

|  |
| --- |

Insert Picture 2 *(Instruction 7)*

|  |
| --- |

Write explanation *(Instruction 7)*

Insert Picture 3 *(Instruction 14)*

|  |
| --- |

Write explanation *(Instruction 14)*

Write explanation *(Instruction 17)*

Insert Picture 4 *(Instruction 20)*

|  |
| --- |

Insert Picture 5 *(Instruction 20)*

|  |
| --- |

Insert Picture 6 *(Instruction 20)*

|  |
| --- |

Insert Picture 7 *(Instruction 20)*

|  |
| --- |

Insert Picture 8 *(Instruction 22)*

|  |
| --- |

Write description *(Instruction 22)*
