# Supplementary material for: Using PyMOL to Understand Why COVID-19 Vaccines Save Lives
Source: J Chem Educ. 2023 Feb 28;100(3):1351–6. doi: 10.1021/acs.jchemed.2c00779 (PMC9999942; doi:10.1021/acs.jchemed.2c00779)
Supplement: Supplementary file 12 — ed2c00779_si_012.docx [file ed2c00779_si_012.docx]

Supporting Information

Using PyMOL to understand why COVID-19 vaccines save lives.

Celia Maya*

Instituto de Investigaciones Químicas (IIQ), Departamento de Química Inorgánica and Centro de Innovación en Química Avanzada (ORFEO-CINQA)

Consejo Superior de Investigaciones Científicas (CSIC) and University of Seville

Avda. Américo Vespucio, 49, 41092 Sevilla (Spain)

* maya@us.es

- **Lab Report – Session 3**

**Lab Report – Session 3**

What are these structures? *(Instruction 3)*

**7v2a**

**7tb8**

**7wpd**

**7czp**

**7czq**

**7jzl.**

***Why vaccines prevent SARS-CoV-2 infections and save hundreds of thousands of lives?***  *(Instruction 4)*
