# Supplementary material for: Using PyMOL to Understand Why COVID-19 Vaccines Save Lives
Source: J Chem Educ. 2023 Feb 28;100(3):1351–6. doi: 10.1021/acs.jchemed.2c00779 (PMC9999942; doi:10.1021/acs.jchemed.2c00779)
Supplement: Supplementary file 14 — ed2c00779_si_014.docx [file ed2c00779_si_014.docx]

Supporting Information

Using PyMOL to understand why COVID-19 vaccines save lives.

Celia Maya*

Instituto de Investigaciones Químicas (IIQ), Departamento de Química Inorgánica and Centro de Innovación en Química Avanzada (ORFEO-CINQA)

Consejo Superior de Investigaciones Científicas (CSIC) and University of Seville

Avda. Américo Vespucio, 49, 41092 Sevilla (Spain)

* maya@us.es

**TEST for assessment**

(Right answers in bold)

1.- How many receptor-binding domains are there in the Spike protein of the SARS-CoV-2 virus?

a) One

b) Two

**c) Three**

d) Four

2.- Choose the correct sentences:

**a) ACE2 is a transmembrane protein that acts as a functional receptor in human cells.**

b) ACE2 is a functional receptor on the coronavirus surface.

c) A RBD domain of the Spike coronavirus protein can interact with the single-helix transmembrane domain of ACE2.

**d) A RBD domain of the Spike coronavirus protein can interact with the catalytic peptidase domain of ACE2.**

3.- Research about Spike protein has demonstrated:

**a) that the antibodies generated with vaccines bind to the receptor binding domain of the Spike Protein**

b) that the antibodies generated with vaccines bind to the ACE2 receptor of the human cells.

c) that the antibodies generated with vaccines kill viruses in the blood

d) that the antibodies generated with vaccines are not capable to stop the disease.

4) Which one or ones of the following groups are contained in the Spike protein of the SARS-CoV-2 virus?

**a) S1 and S2 subunits**

b) a zinc-finger domain

**c) a receptor-binding domain**

d) a catalytic peptidase domain

5) The vaccines to prevent SARS-CoV-2 infection

a) generate antibodies that block the receptors on the cell surface.

**b) generate antibodies that hinder the spike protein union to the human cell receptors.**

**c)** generate antibodies that bind to the white blood cells

d) generate antibodies that go to the nasal cavity and avoid the infection.
